# PalC, One of Two Bro1 Domain Proteins in the Fungal pH Signalling Pathway, Localizes to Cortical Structures and Binds Vps32

**DOI:** 10.1111/j.1600-0854.2007.00620.x

**Published:** 2007-08-13

**Authors:** Antonio Galindo, América Hervás-Aguilar, Olga Rodríguez-Galán, Olivier Vincent, Herbert N Arst, Joan Tilburn, Miguel A Peñalva

**Affiliations:** 1 Departamento de Microbiología Molecular, Centro de Investigaciones Biológicas CSIC Ramiro de Maeztu 9, Madrid 28040, Spain; 2 Departamento de Bioquímica y Genética de Levaduras, Instituto de Investigaciones Biomédicas CSIC Arturo Duperier 4, 28029 Madrid, Spain; 3 Department of Molecular Microbiology and Infection, Imperial College London Flowers Building, Armstrong Road, London SW7 2AZ, UK

**Keywords:** arrestin, *Aspergillus*, endosomes, ESCRT-III, pH regulation, signal transduction, 7-TMD

## Abstract

PalC, distantly related to *Saccharomyces cerevisiae*peripheral endosomal sorting complexes required for transport III (ESCRT-III) component Bro1p and one of six *Aspergillus nidulans*pH signalling proteins, contains a Bro1 domain. Green fluorescent protein (GFP)-tagged PalC is recruited to plasma membrane-associated punctate structures upon alkalinization, when pH signalling is active. PalC recruitment to these structures is dependent on the seven transmembrane domain (7-TMD) receptor and likely pH sensor PalH. PalC is a two-hybrid interactor of the ESCRT-III Vps20/Vps32 subcomplex and binds Vps32 directly. This binding is largely impaired by Pro439Phe, Arg442Ala and Arg442His substitutions in a conserved region mediating interaction of Bro1p with Vps32p, but these substitutions do not prevent cortical punctate localization, indicating Vps32 independence. In contrast, Arg442Δ impairs Vps32 binding and prevents PalC-GFP recruitment to cortical structures. pH signalling involves a plasma membrane complex including the 7-TMD receptor PalH and the arrestin-like PalF and an endosomal membrane complex involving the PalB protease, the transcription factor PacC and the Vps32 binding, Bro1-domain-containing protein PalA. PalC, which localizes to cortical structures and can additionally bind a component of ESCRT-III, has the features required to bridge these two entities. A likely *S. cerevisiae*orthologue of PalC has been identified, providing the basis for a unifying hypothesis of gene regulation by ambient pH in ascomycetes.

Signal transduction is initiated at receptors located in the plasma membrane whose activity is downregulated by signal-induced receptor endocytosis. This is the case when endocytosed receptors follow the endocytic pathway to the lumen of the lysosome/vacuole for subsequent degradation after sorting into the multivesicular (MVB) body pathway (1–3). However, the view that endocytosis equals downregulation has been challenged by reports demonstrating that Tyr kinase, Ser/Thr kinase or seven transmembrane domain (7-TMD) receptors signal from endosomes, with endocytic internalization playing a positive rather than a negative role in signal transduction (4). In pioneering work, Vieira et al. (5) demonstrated that clathrin-dependent endocytosis is required for full epidermal growth factor receptor phosphorylation and activation of, for example, the downstream mitogen-activated protein kinases (MAPK) ERK1 and ERK2. Recently, Gpa1p, the Gα subunit of the heterotrimeric G protein mediating 7-TMD receptor-dependent mating responses in yeast has been shown to signal from endosomes by activating the Vps34p phosphatidylinositol-3 kinase, probably after GTP exchange promoted by the endocytosed receptor (6).

One well studied paradigm where signalling by activated receptors occurs from endosomes involves the β-arrestin- and 7-TMD receptor-dependent activation of MAPK signalling pathways by ‘non-classical’ (i.e. heterotrimeric G protein independent) mechanisms. Beta-arrestin-mediated scaffolding and endocytic internalization of signalling complexes results in persistent, cytosolic MAPK output from endosomes as opposed to transient, nuclear MAPK output resulting from the ‘classical’ G-protein-mediated pathway (7). A mechanistically different paradigm involves signalling from heteromeric Ser/Thr kinase transforming growth factor beta (TGF-β) receptors. In this pathway, R-Smads (key components of the heteromeric complex eliciting transcriptional responses) are anchored to endosomes through the FYVE domain containing/PtdIns (3)*P*-binding protein Smad Anchor for Receptor Activation (SARA). The R-Smad/SARA interaction is destabilized and R-Smads are released upon phosphorylation by activated receptors arriving at early endosomes from the plasma membrane after being sorted and internalized in clathrin-coated vesicles.

In the fungal pH signal transduction pathway, six proteins (denoted PalA, PalB, PalC, PalF, PalH and PalI in *Aspergillus nidulans*) are required for the proteolytic activation of the transcription factor PacC/Rim101 in response to ambient alkaline pH (8,9). Previous work strongly suggested that the pH signalling pathway is organized into two complexes, an ‘upstream’ complex at the plasma membrane involving PalH, PalI and PalF and a ‘downstream’ complex on membranes of the endosomal system involving PalA, the cysteine-protease PalB and PacC/Rim101 (10–12). A key issue is how these seemingly segregated complexes communicate, i.e. how the alkaline pH stimulated plasma membrane complex activates the downstream complex to initiate the proteolytic activation of PacC/Rim101. Evidence strongly indicates that this connection involves endocytic trafficking (10,12). Thus, the fungal pH signalling pathway represents a novel paradigm of a positively acting partnership between endocytosis and signalling (4).

A remarkable feature of the pH signalling pathway is that it shares aspects of both non-classical 7-TMD receptor-dependent and R-Smad pathways. pH regulation resembles G-protein-independent 7-TMD signalling pathways in the positive role that the arrestin-like protein PalF has in transduction of the alkaline pH signal by the 7-TMD receptor protein PalH. Thorough genetic analysis has provided no evidence for involvement of heterotrimeric G proteins in pH signalling (11,13–19) and PalF, a strong interactor of the PalH cytosolic tail, is a positive-acting arrestin-like protein that is ubiquitinated in a PalH- and signal (alkaline pH)-dependent manner (10). Notably, the pH signalling pathway also resembles the TGF-β/R-Smad pathway in that it involves recruitment of the transcription factor (here PacC/Rim101p) to endosomal membranes through PalA (Rim20p in yeasts), an endosomal anchor analogous to SARA. In contrast to SARA, which binds strongly to endosomal membranes through its FYVE domain (20), PalA/Rim20p binds to endosomes through its interactor Vps32p (12,21–23), a subunit of endosomal sorting complex required for transport-III [ESCRT-III (3,24)]. Endosomal sorting complexes required for transport-I, II and III are heteromeric protein complexes acting sequentially in the sorting of membrane proteins into the MVB pathway. Vps32p recruitment to endosomal membranes is dependent on ESCRT-I and ESCRT-II components and on ESCRT-III Vps20 (25–29). Loss of function mutations in ESCRT-I, ESCRT-II, Vps32p and Vps20p genes preclude pH signalling (11,16,17,23,30–32), as would be expected if Vps32p mediates pH signalling when loaded on endosomal membranes.

Prior to this work, PalC (33) was the only *A. nidulans*protein in the pH signalling pathway whose function was completely unknown and which had not been placed in either of the pH signalling protein complexes. PalC orthologues are widely distributed across the fungal kingdom, including in the yeast *Yarrowia lipolytica*(34) and, as suggested here, in *Saccharomyces cerevisiae*and *Ashbya gossypii*. Here, we show that PalC localizes to punctate structures at or near the plasma membrane in a pH-dependent manner. In addition to this cortical localization, PalC binds to ESCRT-III Vps32 and thus has the features expected for a link between the upstream and the downstream pH signalling complexes.

## Results

### Involvement of PalC in PacC processing

**Figure 1:**
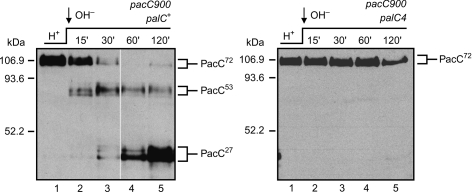
**PalC is absolutely required for pH signalling.**Western blot analysis of Myc-tagged PacC two step proteolytic processing in *palC*^+^ and *palC*4 cells cultured under acidic conditions and shifted to alkaline conditions. The positions of PacC^72^, PacC^53^ and PacC^27^ are indicated. The loss of function mutation *palC4*prevents the signalling protease cleavage converting PacC^72^ to PacC^53^ (and thus prevents processing proteolysis to PacC^27^) (35). Note the previously reported markedly abnormal electrophoretic mobility of the PacC forms in SDS gels, possibly resulting from the highly basic N-terminal zinc-finger region (51).

When wild-type *A. nidulans*cells are transferred from acidic to alkaline pH, full-length PacC^72^ is activated through a two-step proteolytic processing resulting in the conversion of PacC^72^ to the intermediate PacC^53^ and the PacC^27^ final product. *palC*4, a phenotypically null mutation, truncates the protein after residue 365 (33), within the Bro1 domain similarity region (see below). In mutant *palC4*cells, PacC^72^ remains unprocessed upon transfer to alkaline conditions (Figure 1), similarly to mutant *palA^−^*(35) and *palB^−^*(36) cells. Thus, PalC is absolutely required for the pH-dependent proteolytic activation of PacC.

### PalC and Bro1p sequence similarity in the context of the Bro1 domain structure

**Figure 2:**
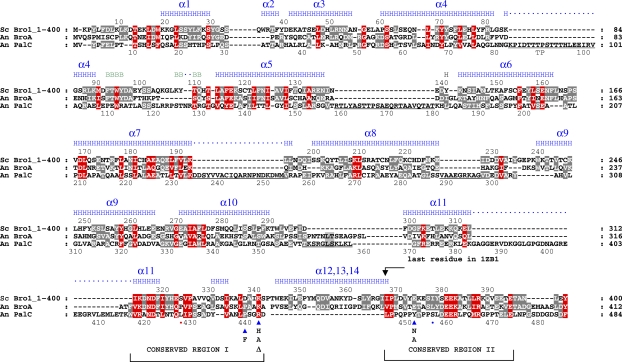
**The Bro1 domain in PalC. Triple amino acid sequence alignment of *Saccharomyces cerevisiae*Bro1p with its *Aspergillus nidulans*orthologue BroA and with PalC.**Shown are Bro1p residues 1 through 400, beyond which no similarity with PalC is discernable. Bro1p residues 1–387 are included in the three-dimensional structure reported by Kim et al. (37). B and H indicate Bro1p residues in β-strands (green) and α-helices (blue), respectively. Note that the positions of secondary structure elements, taken from the authors’ secondary structure assignment shown in the Protein Data Bank entry 1ZB1, are slightly different from those in (37). Underlined PalC residues indicate regions located between predicted α-helical structure. The two conserved regions discussed in the text are bracketed. The position of PalC single-residue substitutions/deletion used in this work is indicated with blue triangles. Also indicated are the positions of PalC truncations corresponding to *palC131*(S455fs, blue dot) and *palC179*(L427stop, red dot) resulting in partial and complete loss of function, respectively (34). Residues conserved in all three proteins (according to the Blosum62 matrix) are highlighted in red, whereas those conserved in only two entries are highlighted in grey. Dashes indicate gaps. Residue numbers are indicated for Bro1p and PalC, above or below the respective sequences.

Figure 2 shows a sequence alignment of a region encompassing the Bro1 domains of the prototype *S. cerevisiae*Bro1p/Vps31p, its *A. nidulans*orthologue BroA and PalC, with indication of secondary structure elements in Bro1p. Sequence similarity is rather conspicuous in several regions within the crystallographically determined Bro1 domain in Bro1p (37) and actually extends ∼30 residues beyond it, strongly supporting our previous observation (34) that PalC contains one such domain, albeit rather distant from those in BroA/Bro1p and its PalA/Rim20p paralogues. The Bro1 domain structure is mostly made of α-helices forming a single 11-helical solenoid (37), with helices 6 through 11 arranged in a tetratricopeptide-repeat-like substructure involving three paired helices and a C-terminal unpaired one (α-helix 11). While a significant proportion of sequence divergence between PalC and Bro1p roughly involves insertions between or at the borders of α-helical regions, the highest similarity is apparent in regions corresponding to Bro1p α-helices. Notable exceptions are two highly conserved regions downstream of Bro1p residue 312 (Figure 2). Conserved region I, partially overlapping with α-helix 11, involves Bro1p residues 313–340, thus including Leu336, Ala338 and Ile339, located at the hydrophobic patch 1 in the Bro1p structure (37). This hydrophobic patch 1 is rimmed by polar, solvent exposed Bro1p residues including Lys340 (Figure 2). Conserved region II (Bro1p residues 367-394) is located downstream of the most C-terminal ordered residue and thus, structural information for it is not available. PalC contains, within this region, a Pro-rich motif with an internal Tyr that is required for pH signalling *in vivo*(34).

One conserved feature of Bro1-domain-containing proteins is their ability to bind the ESCRT-III component Vps32. Kim et al. showed that the hydrophobic patch 1 of Bro1p plays a key role in binding Vps32p, and that the Bro1p Leu336 substitution to Ala blocks this interaction (37). Leu336 is conserved in PalC (Leu438), and a substitution in an adjacent residue (Pro439Phe; Figure 2) results in loss of function (34). Finally, PalC has an Arg (Arg442) in the position corresponding to Lys340, whose deletion or substitution to His results in complete and partial loss of function, respectively (34). Taken together, these data suggested the possibility that PalC binds Vps32 through this region.

### Two-hybrid interactions demonstrate that PalC is linked to ESCRT-III

**Figure 3:**
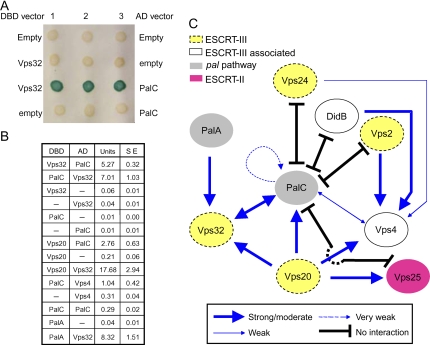
**PalC two-hybrid interactions with ESCRT-III.**A) PalC interacts with Vps32. The indicated GAL4 DNA-binding domain (DBD) and activation domain (AD) combinations were tested in *Saccharomyces cerevisiae*Y187 using the β-galactosidase filter lift assay. B) The indicated DBD and AD protein fusions were tested for two-hybrid interactions using quantitative β-galactosidase assays (given as enzyme units; SE, standard error). The reported PalA–Vps32 interaction is used here as a positive control. C) Network of PalC interactions tested in this work. The scheme summarizes data obtained using β-galactosidase quantitative (Figure 3B) and filter lift (not shown) assays with *S. cerevisiae*Y187 and the -Leu, -Trp, -His, -Ade QSM system (Figure S1) with *S. cerevisiae*AH109. Arrows and ‘T’ symbols denote positive and negative two-hybrid interactions for a given prey-to-bait combination, as indicated. Those combinations that are not indicated have not been tested (Figure S1). Readers should note that while we have tested every possible two-hybrid combination between PalC and ESCRT-III proteins, we have not analysed interactions of all class E Vps proteins included in the diagram with one another. However, each of these ESCRT-III proteins has been validated (i.e. shown to give a positive interaction) with at least one partner in one orientation, and PalC is competent for interactions both as prey and as bait.

We addressed the possibility of PalC/ESCRT-III linkage by systematically testing PalC interaction with all components of ESCRT-III and its accessory AAA ATPase Vps4 using two-hybrid analysis (Figures 3 and S1). This analysis required the characterization of *A. nidulans*orthologues of yeast Did2p [the fungal homologue of charged MVB protein 1 (CHMP1) (38,39)], Vps2p, Vps24p, Vps25p and Vps20p, which will be reported elsewhere. *Aspergillus nidulans*Vps32 has been characterized previously (22). [Note that, as indicated in the legend for Figure 3, we have not systematically tested in two-hybrid assays all the possible interactions of these ESCRT proteins with one another, as has been reported for *S. cerevisiae*(40).]

Two-hybrid assays (Figure 3A,B; see also Figure S1) demonstrated that PalC binds strongly to *A. nidulans*Vps32 when fused to either the DNA binding (bait) or the activation (prey) domains of GAL4. PalC additionally showed two-hybrid interaction with Vps20 (Figure 3B, only one possible orientation tested, involving Vps20 fused to the DNA-binding domain, as fusion to the activation domain is toxic) and, rather weakly, with Vps4. Finally, PalC very weakly interacted with itself (Figure 3B). Figure 3C summarizes the network of two-hybrid interactions tested. The specificity of these interactions was shown by the lack of two-hybrid interaction of bait/prey combinations involving PalC and *A. nidulans*ESCRT-III proteins Vps24 and Vps2, the accessory ESCRT-III protein DidB^Did2^ and ESCRT-II Vps25 [Vps24, Vps2, Vps25 and DidB^Did2^ were competent as two-hybrid interactors with other bait or prey combinations (Figures 3B,C and S1; for DidB^Did2^, our unpublished data)] and by mutational analyses below. As Vps32, DidB^Did2^, Vps24, Vps20 and Vps2 contain a PFAM 03357 Snf7 domain, these experiments additionally show that PalC does not indiscriminately bind to any Snf7-domain-containing protein.

We used β-galactosidase assays for quantitatively estimating the positive interactions (Figure 3B). These showed that the β-galactosidase activity from the interaction between PalC and Vps32 was similar to that between PalA and Vps32, which interact directly (22 and our unpublished data). In contrast, the PalC interaction with Vps20 was only half that obtained with Vps32 and that with Vps4 was markedly weaker (Figure 3B). These assays additionally confirmed the weak, yet significant interaction of PalC with itself (Figure 3B). As Vps20, Vps4 and PalC interact very strongly with Vps32, Vps2 (and DidB) and Vps32, respectively (Figures 3B,C and S1), moderately strong or weak two-hybrid interactions involving these proteins very likely reflect the actual relative strength of the interactions rather than the relative levels of expression of the interacting partners. However, as we have not determined the steady-state levels of baits and preys by Western blot, we cannot rule out the possibility that β-galactosidase assays to some extent reflect differences in expression and/or stability of two-hybrid constructs.

Vps20p interacts directly with Vps32p to form an endosomal-membrane-binding ESCRT-III subcomplex (26) (Figure 3C). In *S. cerevisiae*, Vps4p is also a recognized Vps32p interactor. Thus, we concluded that this apparently promiscuous interactivity of PalC with three different proteins and additionally with itself most likely indicates that PalC interacts directly with at least one component of the Vps20p/Vps32p ESCRT-III subcomplex, thus facilitating the formation of heterooligomeric complexes in which one or more yeast partners bridge an indirect bait–prey interaction. These indirect interactions would be facilitated by the similarity of *A. nidulans*and *S. cerevisiae*proteins. We additionally concluded that the most likely direct partner of PalC in ESCRT-III is Vps32.

**Figure 4:**
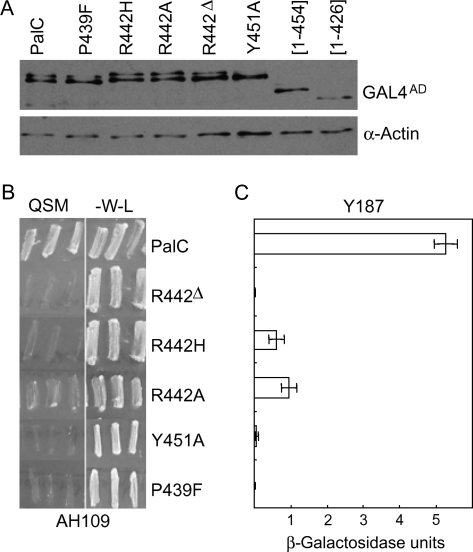
**Single-residue substitutions/deletion within PalC conserved regions I and II largely impair or prevent its interaction with Vps32.**A) Western blot analysis of yeast strains expressing the indicated fusion proteins, which were used as preys in two-hybrid assays. Fusion proteins were detected with anti-HA antibody. Actin was used as a loading control. B) PalC mutations affecting two-hybrid interaction with Vps32, as determined by quadruple selection on -Leu, -Trp, -His, -Ade medium using strain AH109. The experiment was carried out in triplicate. QSM indicates quadruple selection medium. -W-L is medium lacking Tp and Leu. C) PalC mutations affecting two-hybrid interaction with Vps32, as determined by quantitative β-galactosidase assays using strain Y187. Bars indicate standard errors.

We mapped the region of PalC involved in binding Vps32 by testing the effects of PalC single-residue substitutions in two-hybrid assays. While Arg442Δ is phenotypically null *in vivo*, PalC substitutions Tyr451Asn, Pro439Phe and Arg442His (Figure 2) lead to considerable yet slightly incomplete loss of function, as determined using the most sensitive tests (34). In an attempt to exacerbate the phenotype of substitutions involving Arg442 and Tyr451, we constructed Arg442Ala and Tyr451Ala, which were confirmed as leading to loss of function (see below). A panel of single-residue substitutions (Pro439Phe, Arg442His, Arg442Ala and Tyr451Ala), a single-residue deletion (Arg442Δ) and two truncating mutations (after residues 426 and 454) were introduced in the GAL4^AD^::PalC protein fusion and tested against the *A. nidulans*Vps32 bait. Western blot analysis of *S. cerevisiae*strains expressing mutant preys revealed that, with the exception of the truncating mutations, whose steady-state level was reduced relative to wild type (and were thus not considered any further), all other mutations did not markedly affect fusion protein levels (Figure 4A), suggesting that these do not result in major adverse effects in PalC folding. Growth tests on selective media (Figure 4B) and β-galactosidase assays (Figure 4C) showed that PalC single-residue mutations largely impaired (Arg442His and Arg442Ala) or prevented (Pro439Phe, Tyr451Ala and Arg442Δ) the two-hybrid interaction. Of note, Arg442Ala and Arg442His reduced β-galactosidase activity to ∼18 and ∼11% of the wild type, respectively, which contrasts with the total lack of activity using Arg442Δ, despite the similar steady-state levels of these three mutant PalC preys. The partial and complete loss of function phenotypes of Arg442His and Arg442Δ, respectively, in two-hybrid assays correlate with the phenotypic effects of the corresponding mutant alleles *in vivo*(34) (see above). These data strongly support the above conclusion that the PalC–Vps32 interaction is specific and point to the conserved regions containing Arg442, Pro439 and possibly Tyr451 (Figure 2) as likely candidate(s) that mediate, at least in part, this specificity. The Bro1 region corresponding to the environs of PalC Tyr451 is not included in the crystal structure. However, as discussed above, Pro439 and Arg442 are within a region that in Bro1p is involved in Vps32p binding (Figure 2).

### PalC is a direct interactor of Vps32

**Figure 5:**
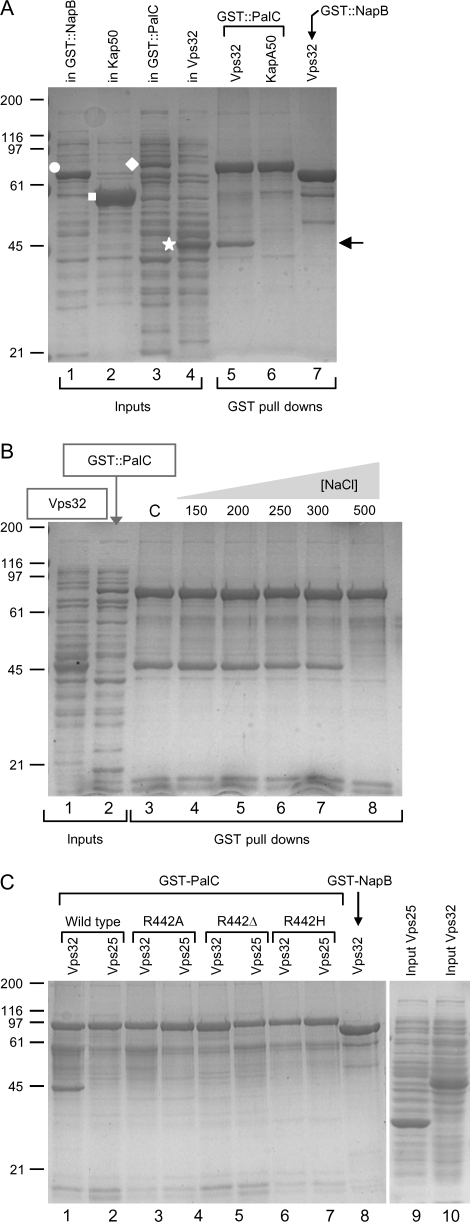
**PalC interacts directly with Vps32 and this interaction involves Arg442 within conserved region I.**A) GST-PalC (white diamond) and zz-Vps32 (white star) fusion proteins expressed in *E. coli*copurify on glutathione–Sepharose beads (GST pull downs) after mixing the corresponding bacterial extracts (lanes labelled ‘in’, inputs). KapA^50^ (white square) is a 50-kDa truncated version of the *A. nidulans*KapA importin α lacking the auto-inhibitory domain. NapB^Vps75^ (white circle) is the *A. nidulans*homologue of *Saccharomyces cerevisiae*Vps75p. The closed arrow indicates the mobility of zz-Vps32 copurifying with GST-PalC. B) GST–PalC interaction with zz-Vps32 resists NaCl up to 300 mm but is disrupted by 500 mm NaCl. C) zz-Vps25 does not copurify with GST-PalC, whose interaction with zz-Vps32 is prevented by Arg442Ala, Arg442His and Arg442Δ. In A and C, the NaCl concentration was 200 mm.

To determine whether the PalC–Vps32 interaction is direct and to rule out the possibility that the presence of N-terminal Gal4p DNA binding and activation domains in Vps32 two-hybrid assay fusion proteins alters the interactive properties of Vps32, we bacterially expressed a glutathione S-transferase (GST)::PalC fusion protein for pull-down experiments with glutathione–Sepharose beads (Figure 5A). GST::PalC efficiently and specifically pulls down zz-Vps32 from a bacterial extract (Figure 5, lane 5). (zz indicates a tandem repetition of a ‘z’ synthetic immunoglobulin-G (IgG)-binding domain of protein A). This interaction is resistant to relatively high ionic strength, as it is prevented only by 0.5 m NaCl but not by 0.3 m NaCl or salt concentrations below that (Figure 5B). In contrast, GST::PalC did not pull down the unrelated protein KapA^50^ (Figure 5A, lane 6) nor did the unrelated GST-NapB fusion protein control pull-down Vps32. Because GST::PalC does not pull down the ESCRT-II component Vps25 either (Figure 5C, lines 1 and 2), we conclude that PalC interacts specifically and directly with Vps32. Under conditions in which glutathione–Sepharose beads were not saturated with GST::PalC (data not shown), quantitative analysis of digitized images indicated that 0.9 mol of zz-Vps32 was pulled down per mole of GST::PalC.

We next expressed single-residue mutant GST::PalC fusion proteins carrying the amino acid substitutions/deletion used in the two-hybrid analyses detailed above. After bacterial overexpression, we obtained sufficient solubility only for Arg442Ala, Arg442His and Arg442Δ GST::PalC fusion proteins. Under standard conditions, all three mutations prevented Vps32 binding (Figure 5C) (which represents additional evidence for the specificity of the interaction).

### PalC-GFP subcellular localization as a function of ambient pH

**Figure 6:**
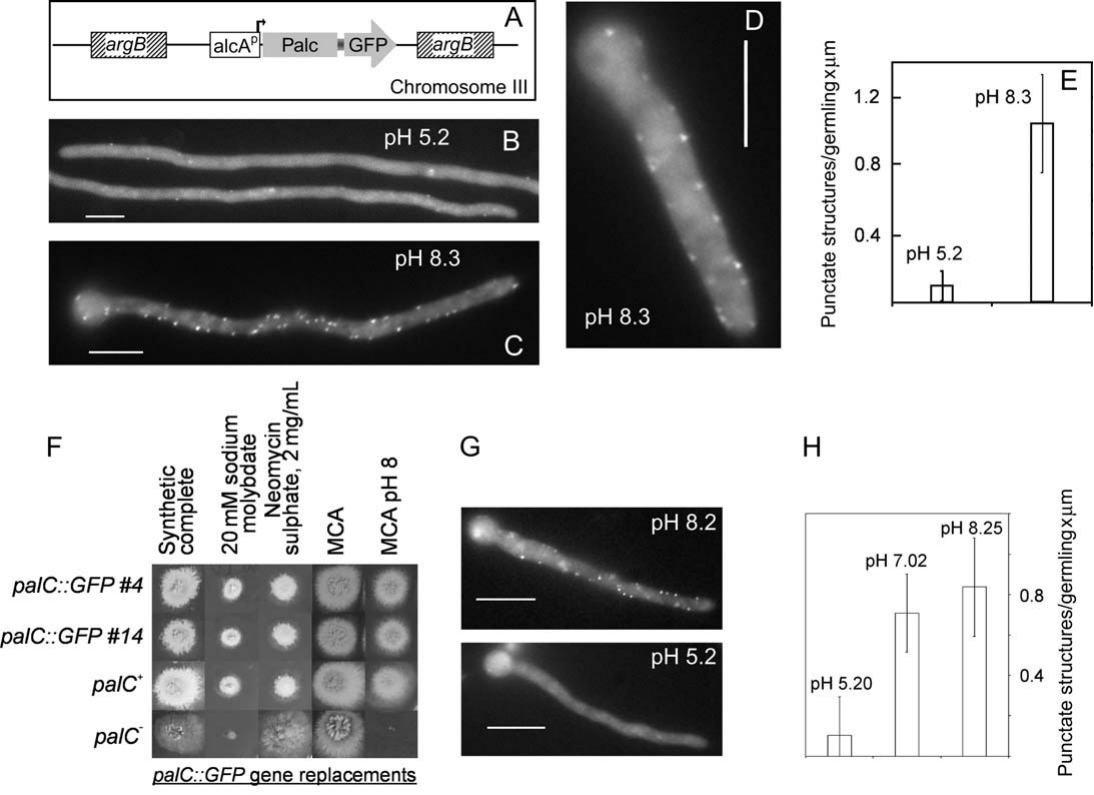
**PalC-GFP localizes to the cytosol and to peripheral punctate structures in a pH-dependent manner.**A) An *alcA*^p^::*PalC-GFP*transgene was introduced in single copy at the *argB*locus. Spores were germinated under inducing conditions at acidic pH and transferred for 30 minutes to repressing conditions at either acidic (B) or alkaline (C and D) pH before imaging GFP fluorescence. B) Subcellular localization of PalC-GFP upon transfer to acidic conditions. A few peripheral punctate structures are noticeable, a situation which was also seen in control germlings imaged before the transfer to repressing conditions (not shown). C) Subcellular localization of PalC-GFP upon transfer to alkaline conditions. Note the marked increase in the abundance of punctate structures. D) A short germling showing the cortical localization of PalC-GFP puncta. E) Quantification of punctate structures in germlings containing the *alcA^p^*-driven transgene shifted to acidic or alkaline conditions. F–H) Phenotypic characterization of *palC-GFP*gene-replaced strains and subcellular localization of the fusion protein expressed at physiological levels. F) Diagnostic growth tests showing that two independent clones in which the resident *palC*open reading frame has been replaced by an open reading frame encoding a PalC-GFP fusion are indistinguishable from the wild type. Acidity-mimicking loss of function mutations in *palC*typically result in hypersensitivity to sodium molybdate and alkaline pH and resistance to neomycin. MCA is *Aspergillus*complete medium. G) Strains were cultured at acidic pH and shifted to the indicated pH conditions for 30 minutes before GFP imaging. No punctate structures were seen in untagged control strains at either pH (not shown). H) Quantification of punctate structures in germlings shifted to acidic, neutral or alkaline conditions. Because significant pH signalling takes place under neutral conditions, PalC-GFP punctate structures are nearly as abundant upon transfer to neutral as to alkaline conditions. Bars, 5 μm.

We constructed transgenes encoding PalC tagged with GFP at the N- or C-terminus, under the control of the strong, ethanol inducible and glucose repressible *alcA*(alcohol dehydrogenase) gene promoter and introduced them in single copy at the *argB*locus (Figure 6A). N-terminal GFP attachment abolished PalC function, as determined by the inability of the fusion protein to complement the *palC4*loss of function mutation (data not shown). In contrast, PalC-GFP was phenotypically indistinguishable from untagged PalC in its ability to complement *palC4*under *alcA*^p^ inducing conditions (Figure S2). A strain expressing this fusion protein was thus used in epifluorescence microscopy.

Preliminary experiments revealed that in germlings cultured under inducing conditions PalC-GFP localized to the cytosol and to punctate structures, contrasting with the uniform nucleocytosolic distribution of control GFP (see below). To address the possibility that this subcellular localization changes in a pH-dependent manner, germlings were cultured overnight at acidic pH under inducing conditions and subsequently shifted to repressing conditions in media adjusted to acidic (pH 5.2) or alkaline (pH 8.3) pH. Epifluorescence microscopy after an additional 30-minute incubation revealed that transfer to alkaline pH resulted in a marked increase in the number of PalC-GFP punctate structures (Figure 6C–E) compared with cells shifted to acidic pH conditions (Figure 6B,E). These punctate structures were clearly cortical (Figure 6D). No such structures were observed in a control strain expressing GFP alone under either pH condition (Figure S3). Experiments with an untagged strain ruled out the possibility that puncta involve auto-fluorescent structures promoted by the alkaline pH shift (not shown). Finally, shifting cells to neutral (pH 7) conditions, where significant pH signalling takes place, also led to a marked increase in the number of punctate structures (not shown, see also below).

To rule out the possibility that this punctate localization of PalC-GFP was an artefact resulting from overexpression, we used the procedure of Osmani et al. (41) to GFP-tag PalC at the C-terminus after gene replacement (Figure 6F–H). This enabled imaging of PalC-GFP expressed at physiological levels and minimized the possibility that the GFP-tag interferes with PalC folding (thereby leading to fusion protein aggregates) by introducing a (Gly-Ala)_5_ poly-linker between the two moieties. Strains carrying the chimaera were phenotypically indistinguishable from the wild type (Figure 6F) and expressed a GFP fusion protein of the expected size, as determined by Western blot (see below). Four independent strains examined by epifluorescence microscopy showed a punctate distribution similar to that seen in the *alcA*^p^-driven strains, although the intensity of the spots relative to the cytosolic fluorescent background was reduced (data not shown), in agreement with the expectedly lower levels of the fusion protein expressed in these strains. Notably, gene-replaced strains behaved as those carrying the *alcA*^p^-driven transgene in that the abundance of punctate structures was markedly increased on transfer to neutral and alkaline pH (Figure 6G,H).

In summary, punctate structures imaged in GFP-tagged PalC strains markedly increased in number under conditions activating the pH signalling pathway, were never observed when GFP was expressed without an attached PalC moiety and were seen equally in gene replaced and in PalC-GFP overexpressing strains, strongly suggesting that they represent one physiological subcellular localization of PalC.

### Substitutions affecting Vps32 binding and their effect on PalC-GFP localization

**Figure 7:**
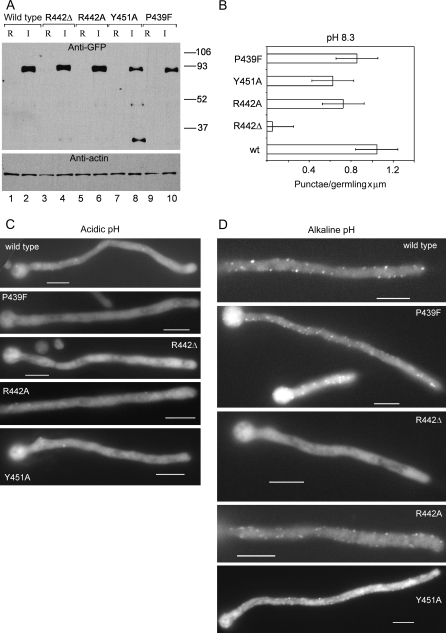
**Arg442Δ but not other mutations impairing or preventing PalC interaction with Vps32 prevents the alkaline pH-dependent increase in punctate subcellular localization of PalC-GFP.**A) Western blot analysis of mutant PalC-GFP proteins. Mycelia of strains carrying wild type or mutant *alcA*^p^::PalC-GFP transgenes, as indicated, were precultured under derepressing, noninduced conditions and shifted to repressing (R) or inducing (I) conditions before proceeding to protein extraction and Western blot analyses using anti-GFP antibodies or anti-actin as loading control. B) Quantification of PalC-GFP punctate structures upon shifting the indicated strains to alkaline pH as in legend to Figure 6. C and D) Representative images of PalC-GFP localization of wild type and mutants under alkaline conditions. Bars, 5 μm.

We introduced, in the *alcA*^p^::*palC*-*GFP*transgene, mutations leading to PalC Arg442Ala, Pro439Phe or Tyr451Ala substitutions or to the Arg442Δ in-frame deletion. None of these mutant transgenes complemented a phenotypically null *palC4*allele in plate tests, which demonstrates that Arg442Ala and Tyr451Ala substitutions result in a loss of function phenotype (Figure S2). Western blot analyses showed that, under inducing conditions, Arg442Ala and Arg442Δ mutant transgenes synthesized full-length PalC-GFP proteins with steady-state levels similar to the wild type (Figure 7A, compare anti-GFP lanes 4 and 6 with lane 2). In contrast, Tyr451Ala and Pro439Phe PalC-GFP levels were lower (Figure 7A, lanes 8 and 10). Detection of degradation bands in the Tyr451Ala mutant indicates that this particular sequence change leads to decreased protein stability in *Aspergillus*.

Figure 7B–D shows that all three single-residue substitution mutants showed pH-dependent peripheral PalC-GFP puncta, although the cytosolic background was noticeably more intense with the Tyr451Ala mutant (note that a major degradation band from this mutant corresponds to the GFP moiety largely devoid of PalC sequences, Figure 7A, lane 8). In marked contrast, alkaline pH-induced punctate structures were hardly seen in the Arg442Δ mutant (Figure 7B,D). Pro439Phe, Tyr451Ala and Arg442Ala prevent or largely impair the PalC–Vps32 interaction (Figures 4 and 5). Thus, the lack of effect of these mutants on the localization of PalC to punctate structures strongly suggests that this localization is Vps32 independent. In contrast, the Arg442Δ deletion, also preventing interaction with Vps32 (Figures 4 and 5), virtually abolishes PalC punctate localization. These data strongly indicate that Arg442Δ must be affecting another (Vps32 independent) aspect(s) of PalC function mediating its recruitment to cortical structures. We speculate that, by introducing Asp443 in position 442 and additionally shifting the position of residues downstream of this position, deletion of Arg442 might impede Vps32 binding while simultaneously distorting structure or relative orientation of a functional domain of PalC mediating such recruitment. The marked effect of Arg442Δ on PalC-GFP subcellular localization further supports the contention that punctate structures represent one physiological localization of PalC.

### PalC localization is dependent on the 7-TMD receptor PalH but not on PalA

**Figure 8:**
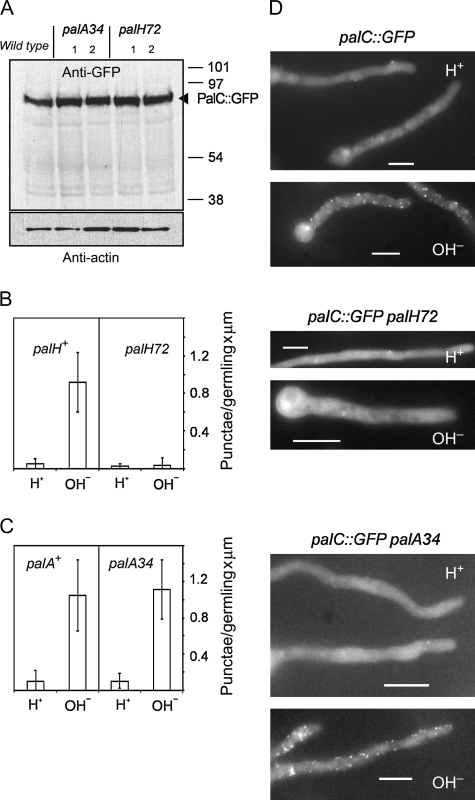
**PalH-dependent and PalA-independent localization of PalC to cortical punctate structures.**Strains carrying the gene-replaced *palC-GFP*allele in combination with *palA34*or *palH72*were compared with *palC-GFP*wild-type controls. A) Western blot analyses, using anti-GFP and anti-actin antibodies, of protein extracts from the wild type and from mutant strains (two clones with the indicated genotypes were analysed among the progeny of each cross). B and C) Quantification of PalC-GFP punctate structures upon shifting the indicated strains to alkaline pH. D) Representative images of PalC-GFP localization in the wild type and in the indicated mutants under acidic and alkaline conditions. Bars, 5 μm.

As PalC localization to cortical punctate structures appears to be independent of Vps32, but dependent on ambient pH, we tested whether this localization requires the 7-TMD receptor and likely pH sensor PalH. The null *palH72*mutation truncates the 760 residue after residue 12 (S. Negrete-Urtasun, E. A. Espeso, H. N. A. and M. A. P., Imperial College London and Centro de Investigaciones Biológicas Madrid, unpublished results). We constructed by meiotic recombination a strain carrying *palH72*, in which PalC-GFP is the only functional PalC, for comparison with the corresponding *palH*^+^ strain. The genotype of this strain was confirmed by direct sequencing of the mutant *palH72*allele. Western blotting demonstrated that full-length PalC-GFP was synthesized irrespective of the presence or absence of the *palH*72 mutation (Figure 8A). Both *palH*^+^ and *palH72*strains showed predominant cytosolic fluorescence under acidic conditions. However, when cells were shifted to alkaline pH, *palH72*prevented the characteristic wild-type PalC-GFP localization to cortical puncta (Figure 8B,D).

We similarly addressed whether PalC-GFP localization was affected by *palA34*, a mutation truncating the 897 residue PalA protein after residue 32 (our unpublished data), which thus represents a null allele. In contrast to *palH72*, *palA34*did not prevent the ambient pH-dependent localization of PalC-GFP to cortical puncta (Figure 8C,D). This experiment is relevant because PalA, like PalC, contains a Bro1 domain and PalA is the prototypic component of the downstream signalling complex and an interactor of the signalling proteolysis substrate PacC^72^(10,12,21,22). Thus, these experiments demonstrate that PalC is recruited to cortical sites in an ambient pH- and 7-TMD receptor PalH-dependent manner, but that its recruitment is PalA independent, strongly suggesting that PalC plays a role downstream of the plasma membrane signal reception complex and upstream or in concert with the PalA-containing complex.

### Characterization of Vps32- and PalC-containing compartments

**Figure 9:**
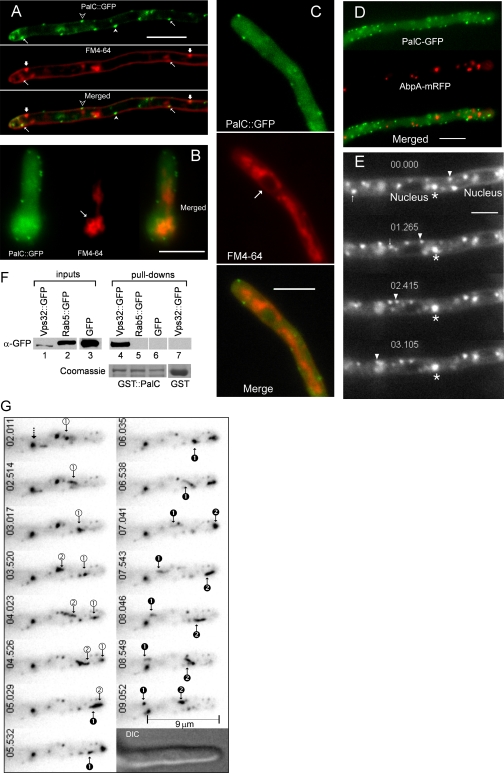
**Characterization of PalC-GFP and Vps32-GFP-containing compartments.**A) PalC-GFP punctate structures and FM4-64-stained plasma membrane and associated cortical structures stained shortly after dye loading. Examples of cortical and nearly cortical PalC-GFP punctate structures that do not associate with cortical FM4-64 puncta are indicated by empty and filled arrowheads, respectively. Thick arrows indicate two examples of FM4-64 cortical structures that do not associate with PalC-GFP. Thin arrows indicate two (rare) examples where PalC-GFP and FM4-64 cortical structures are closely associated. B and C) PalC-GFP punctate structures do not associate with endomembranes stained with FM4-64. B) One of several vacuoles labelled by FM4-64 after a 45-minute chase (see text) in the swelled basal conidiospore is arrowed. C) Endomembranes stained with FM4-64 after a 45-minute chase include a network of mitochondria and endoplasmic reticulum (ER). One nucleus showing labelling of its ER-associated membrane is arrowed. D) PalC-GFP punctate structures do not associate with AbpA-mRFP-labelled actin patches. E) Motile FM4-64 early endosomes filmed within 5 minutes after dye loading in an incubation chamber at 28°C. Frames were taken from Movie S2, which was made using the stream acquisition feature of the MetaMorph Universal Imaging software with 0.1 seconds exposures. Elapsed time is indicated in seconds:milliseconds. One of the several endosomes moving very near the cortex is indicated with an arrowhead. A second endosome moving in the opposite direction and stopping in the vicinity of a nucleus is arrowed. A larger, static endosome is indicated with an asterisk. Bar, 5 μm. F) GST-PalC pulls down Vps32-GFP but not GFP-Rab5 or GFP from *Aspergillus nidulans*protein extracts from cells expressing the indicated proteins. Glutathione S-transferase does not pull down Vps32-GFP. Glutathione–Sepharose bound proteins were run in twin 10% polyacrylamide gels, one of which was analysed by Western blot using an anti-GFP antibody (α-GFP), whereas the second was stained with Coomassie Blue to show the amounts of GST-PalC and GST baits used in the different lanes. G) Motile endosomes labelled with Vps32-GFP. Frames, shown in inverted contrast, were taken from Movie S3, which should be consulted to get a more accurate record of endosome motility and trajectories. The movie was made using the stream acquisition feature of MetaMorph with 0.5 seconds exposures and a 2 × 2 binning. Elapsed time is indicated in seconds:milliseconds. Endosomes showing retrograde and anterograde movement (two of each class) are indicated with arrowed numbers. Note that subapical ‘black’ endosome 2 appears to be formed after receiving traffic from ‘white’ endosomes 1 and 2 moving in anterograde direction. The dotted arrow indicates a static endosome, which may be used as reference.

PalC-GFP is recruited to cortical punctate structures very rapidly after a pH shift, within the time required to mount germlings on microscopy slides and start fluorescent image acquisition. Using time-lapse fluorescence microscopy, we determined that these structures are static (Movie S1). We attempted a preliminary characterization of these cortical sites where PalC is recruited using the lipophilic fluorescent dye FM4-64. Within a 5-minute chase after a cold loading of FM4-64 (42), the dye labels the plasma membrane and cortical punctate structures that possibly represent sites of lipid internalization. Double-label experiments demonstrated that PalC-GFP cortical structures colocalize or closely associate with the plasma membrane (Figure 9A, empty and filled arrowheads, respectively). FM4-64 and PalC-GFP cortical punctate structures occasionally overlap (Figure 9A, thin arrows), although in a majority of cases they do not associate (e.g. Figure 9A, thick arrows). After a 45-minute chase, FM4-64 is found in vacuolar membranes (Figure 9B, arrow) and in a network of endomembranes containing mitochondria and endoplasmic reticulum (Figure 9B,C) (42). PalC-GFP-containing structures do not associate with either (Figure 9B,C; the position of a nucleus in panel C is indicated with an arrow). As in *S. cerevisiae*, the *A. nidulans*actin cytoskeleton involves actin cables and cortical patches (43). To determine whether PalC-GFP structures associate with cortical actin patches, we made double-labelling experiments with AbpA-monomeric red fluorescence protein (mRFP). AbpA (the orthologue of yeast Abp1p) is a prototypic marker of these sites (L. Araujo-Bazán, M. A. P. and E. Espeso, submitted). PalC-GFP and AbpA-mRFP do not colocalize. AbpA patches are slightly subcortical and most of them are seen in a different focal plane than PalC-GFP (Figure 9D).

Early endosomes can be visualized after labelling germlings growing in an incubation chamber with a short pulse of FM4-64 (42). The tubular, polarized cells of *A. nidulans*are usually several tens of microns in length. Figure 9E (which should be consulted together with Movie S2) shows that, as reported previously (42), FM4-64-labelled early endosomes of *A. nidulans*are cytosolic structures that move bidirectionally along the cells at a speed of 2–3 μm/seconds, almost certainly using microtubule-dependent motors. Corroborating evidence that these rapidly moving structures are indeed early endosomes moving on microtubules has been obtained by showing that GFP-Rab5 localizes to structures that move at similar rates and that their movement is prevented by the microtubule depolymerising drug benomyl (M. A. P., J. F. Arenza and J. M. Rodríguez, Centro de Investigaciones Biológicas, Madrid, unpublished data). Thus, as cortical PalC-GFP structures are static, they cannot represent early endosomes. To obtain further evidence that the biogenesis of PalC-GFP punctate structures does not involve the endosomal system, we constructed strains expressing PalC-GFP and carrying deletion alleles of the genes encoding the early endosomal Rab5 GTPase (J. F. Arenza and M. A. P., unpublished data) and the ESCRT-III-associated protein DidB (our unpublished data). [The DidB yeast orthologue Did2p regulates the function of the AAA ATPase Vps4p on ESCRT-III and *did2Δ*leads to the formation of abnormal vesicular tubular endosomes (39,44)]. Neither of the above *A. nidulans*deletions affected the relative density or the intensity of PalC-GFP punctate structures (data not shown). Thus, we conclude that PalC-GFP structures are not endosomes. However, we note that *A. nidulans*microtubules are often subcortical and eventually make contacts with the cell cortex (e.g. 45,46). This proximity to the cortex would facilitate a hypothetical, transient interaction between PalC-GFP-containing structures and endosomes. One example of an early endosome moving in the proximity of the cell cortex is shown in Figure 9E and Movie S2.

We next addressed the subcellular localization of Vps32. In *S. cerevisiae*, deletion of *VPS32*is viable. In contrast, deletion of *A. nidulans*Vps32 is highly deleterious (A. M. Calcagno, M. A. P. and H. N. A., Imperial College, London, and Centro de Investigaciones Biológicas, Madrid, unpublished data). As fusion of GFP to *S. cerevisiae*ESCRT-III proteins prevents their function (44), the finding that C-terminal mRFP tagging of Vps32 severely impairs growth of *A. nidulans*strains where the fusion protein is the only source of Vps32 was not unexpected (data not shown). However, it has been reported that C-terminal GFP tagging of the human Vps32 homologue hSnf7-1 does not preclude its binding to endosomal membranes (which takes place through the N-terminal half of the protein) (47). Most importantly, C-terminal fusion to mRFP does not affect *S. cerevisiae*Vps32p localization to endosomal membranes and does not prevent the ambient pH-dependent processing of Rim101p (12). Thus, we analysed the subcellular localization(s) of Vps32-GFP and Vps32-mRFP in *A. nidulans*strains containing a resident wild-type gene.

We used the GST::PalC bait in pull-down experiments with *A. nidulans*protein extracts from a strain where expression of Vps32-GFP was driven by *alcA^p^*(Figure 9F). Strains where GFP-Rab5 or GFP expression was also driven by *alcA^p^*were used as controls (*Materials and Methods*). GST::PalC already defined above efficiently pulled down Vps32-GFP from *A. nidulans*extracts, but did not pull down the unrelated control proteins GFP-Rab5 or GFP (Figure 9F). In addition to representing further corroborating evidence of the interaction between PalC and Vps32, this experiment shows that Vps32-GFP is competent for PalC binding.

Full promoter induction in the presence of ethanol resulted in Vps32-GFP accumulation in highly fluorescent, relatively large immotile structures, which were often adjacent to vacuoles (data not shown). We reasoned that these structures were probably induced after overexpression because overexpressed hSnf7-1-GFP accumulates on the limiting membranes of enlarged endosomes in human embryonic kidney cultured cells (47). To reduce Vps32-GFP expression markedly, we took advantage of the low levels of transcription that *alcA^p^*shows under noninducing, derepressing conditions, in the presence of 0.1% w/v fructose as sole carbon source (48). Under these conditions, the intensity of the GFP signal, although relatively weak, was nevertheless sufficient to allow epifluorescence detection of the fusion protein. Notably, time-lapse experiments demonstrated that Vps32-GFP localizes to highly motile specks that closely resemble motile FM4-64- and Rab5-GFP-labelled early endosomes in their cytosolic localization and in that they move in either direction and at a similar speed (Figure 9G; frames were taken from Movie S3, which should be additionally consulted). For example, the four specks arrowed in Figure 9G move at rates approximately ranging between 2.2 and 2.7 μm/second. These rates are consistent with involvement of microtubule-dependent motors. As relatively long (0.5 seconds) exposure times were used because of the weak fluorescent signal, moving structures form comet-like tails. As expected for vesicles moving on microtubules, their trajectory frequently brings Vps32-GFP-containing structures in the proximity of the cell cortex, (Movie S3). Vps32-GFP additionally localizes to relatively static and larger cytosolic structures (one is arrowed as reference mark in Figure 9G).

Vps32-mRFP was expressed under the control of the *Vps32*promoter. The fact that this reporter localized to static cytosolic structures and to motile specks indistinguishable from those seen with Vps32-GFP (data not shown) strongly indicated that these structures represent a *bona fide*localization of Vps32.

### YGR122w is a likely S. cerevisiae PalC orthologue

We have previously failed to identify a PalC orthologue in *S. cerevisiae*(34). In view of the above data demonstrating that PalC is a Vps32-interacting protein, we reinspected the *S. cerevisiae*proteome for putative candidates simultaneously containing the conserved region involved in Vps32p binding and the C-terminal di-aromatic residue motif characterizing PalC family members (34). The *YGR122w*product met the above criteria, as did its syntenic *A. gossypii*homologue AAR081Cp (Figure S4). Despite the close relationship between these yeasts, YGR122w and AAR081Cp showed limited amino acid sequence identity (BLASP score 7.9 × 10^−24^, 26% identity in a 344 residue overlap containing 11% gaps), which explains our previous failure to detect a PalC orthologue in *S. cerevisiae*(*S. cerevisiae*and *A. gossypii*are more closely related to each other than to any filamentous fungus). YGR122w is a demonstrated Vps32 two-hybrid interactor (49,50). Most notably, YGR122w is involved in pH signalling (16) and, like other pH signalling *RIM*genes, is required for activation of the yeast PacC orthologue Rim101p (16,17). Additionally, we showed (Figure S5) that *ygr122wΔ*results in Li^+^ hypersensitivity, a phenotypic characteristic of all pH signalling *rim*mutations (21). We conclude that YGR122w is a likely PalC orthologue, although attempts to detect crosscomplementation of either *ygr122w*-associated yeast Li^+^ hypersensitivity by overexpressed PalC (Figure S5) or of the *A. nidulans*pH regulatory null *palC185*phenotype by overexpressed YGR122w (not shown) were unsuccessful, a failure that was not completely unexpected because the yeast pH signalling *RIM*pathway is unable to mediate heterologous activation of PacC in *S. cerevisiae*(51).

## Discussion

This work represents a major advance in understanding the fungal ambient pH signalling pathway by providing new insights into the molecular function of PalC, thus far the most enigmatic among the six dedicated proteins involved in ambient pH signal transduction. We demonstrate: (i) that PalC localizes to cortical punctate structures in an ambient pH- and 7-TMD receptor PalH-dependent manner, and thus, plays a role downstream of the plasma membrane signalling complex; the conclusion that these structures represent a site where PalC plays its role is additionally buttressed by the finding that a phenotypically null Arg442 single-residue deletion prevents PalC localization to these punctate structures at any ambient pH, without affecting protein stability; (ii) that the physiological role of PalC requires another functional feature, in addition to its ability to localize to these structures. This is shown by the strong loss of function phenotype resulting from single-residue substitutions, which do not affect PalC punctate cortical localization to any significant extent; (iii) that three such single-residue substitutions are located within a sequence motif resembling a hydrophobic patch in Bro1p, which mediates binding to Vps32p. We demonstrate by two-hybrid and recombinant protein pull-down assays that PalC binds Vps32 and that these single-residue substitutions prevent this binding. Thus, an additional functional feature of PalC resides in its ability to bind Vps32 and PalC recruitment to cortical punctate structures appears to be Vps32 independent; (iv) that this punctate localization is independent of PalA, placing PalC function upstream of or in concert with the downstream pH signalling complex. This agrees with our previous conclusion that PalC acts, like PalB and PalA, downstream of the PalH 7-TMD receptor-dependent ubiquitination of the arrestin-like protein PalF (10) and (v) that the *YGR122w*product is the likely PalC orthologue, the only component of the *pal*pathway previously considered to be missing in *S. cerevisiae*(34), thus providing the basis for a unifying hypothesis of pH regulation in ascomycetes.

The N-terminal 442 residues of PalC show convincing similarity to the crystallographically determined Bro1 domain in Bro1p (34 and this work). Here, we demonstrate that PalC shares with Bro1p and other Bro1-domain-containing proteins the ability to bind the ESCRT-III component Vps32. Loss of function mutations *palC90*, *palC113*, *palC162*and *palC87*resulting in single residue changes Pro439Phe, Arg442His, Arg442Δ and Tyr451Asn, respectively (34), involve residues located within two regions notably conserved between Bro1p and PalC. Region I in Bro1p, which contains Pro439 and Arg442 counterparts, contributes several residues to a patch involved in Vps32p binding. Pro439Phe, Arg442His and Arg442Δ among the above changes and the engineered substitution Arg442Ala (also leading to *palC*loss of function) largely prevent or abolish Vps32 binding, as determined by two-hybrid and/or pull-down assays, without noticeably affecting protein stability. This strongly indicates that the interactive role of this region is conserved in PalC and demonstrates that the ability to bind Vps32 is a crucial aspect of PalC function.

Tyr451 in PalC is within the less conserved region II, which is not included in the crystal structure reported by Kim et al. (37). Like Tyr451Asn, Tyr451Ala (this work) results in *palC*loss of function. Tyr451Ala appears to lead to protein instability, which is noticeable when the corresponding fusion protein with GFP is expressed in *A. nidulans*. However, the finding that, in *S. cerevisiae*, the Tyr451Ala substitution abolishes the two-hybrid interaction of a PalC prey with Vps32 without markedly lowering protein levels might suggest that, in PalC, residues beyond the crystallographically determined Bro1 domain contribute to Vps32 binding either directly or indirectly (e.g. by buttressing the proper folding of the Bro1 domain).

In two-hybrid assays, PalC additionally interacts with Vps20, the only reported Vps32 partner in the ESCRT-III subcomplex required for endosomal membrane association (26). Although the relatively weaker two-hybrid interaction of PalC with Vps20 compared with Vps32 suggests that this interaction is probably indirect (Vps32 mediated), we cannot exclude the possibility of a direct PalC–Vps20 interaction. Marked insolubility of Vps20 in our bacterial expression system precluded testing this possibility. In contrast, PalC does not bind either Vps24 or Vps2, the components of the ‘peripheral’ subcomplex required for the Vps4-dependent dissociation of ESCRT-III (26). This apparently stringent specificity for Vps32 and Vps20 agrees with the fact that while these ESCRT-III subunits are absolutely required for pH signalling (11,16,23)*vps2Δ*or *vps24Δ*mutations result in constitutive activation of the pathway (11), presumably by preventing the competition of Vps2p and Vps24p with pH signalling proteins to bind Vps32.

Data in *S. cerevisiae*strongly indicate that membrane recruitment of Vps32/Vps20 plays a crucial role in pH signalling, which requires, in addition to Vps32p and Vps20p, all components of ESCRT-I and ESCRT-II (11,23). Remarkably, *vps2Δ*or *vps24Δ*mutations are able to suppress the pH regulatory phenotype resulting from null ESCRT-I mutations but are unable to suppress *vps20Δ*, *vps32Δ*, *vps36Δ*or *vps25Δ*. This observation may now be revisited in view of the recently reported ability of ESCRT-II to bind endosomal membranes independently of ESCRT-I through a split pH domain present in Vps36p (28). Vps20 binds ESCRT-II through Vps25, and this interaction is conserved in *Aspergillus*(Figure 3). Thus, Vps20 and Vps32 associate with membranes in three ways: (i) through Vps20, which is myristoylated (26); (ii) through direct Vps32 binding to membranes (47) and (iii) through interaction of Vps20 with ESCRT-II (52).

In ascomycetes, three proteins interact through their Bro1 domains with Vps32. These are, in addition to PalC (and its almost certain orthologue YGR122w), PalA/Rim20p (22,23) and Bro1p, the prototypical Bro1-domain-containing protein acting at a late step in the MVB pathway (37,53,54). As Bro1p and Rim20p/PalA play a dedicated role in MVB sorting and pH regulation, respectively (21,55), a conundrum is how the ESCRT-III complex can be engaged in *a priori*mutually exclusive interactions with different Bro1-domain-containing proteins.

One likely explanation involves the multimeric (56), suggested lattice/cage structure of ESCRT-III (57,58), which would provide multiple anchoring points for different partners. This agrees with the partial gain-of-function pH signalling phenotype resulting from *vps2Δ*or *vps24Δ*deletion (11), as the Vps2p/Vps24p subcomplex binds to Vps20p/Vps32p (56) and thus would compete with other Vps32 interactors. In addition, Boysen and Mitchell (12) reported the highly suggestive finding that MVB and pH signalling complexes can be spatially segregated on different endosomal domains. In *S. cerevisiae*, the PalA orthologue and Vps32p-binding protein Rim20p is recruited to endosomes under conditions activating the signalling pathway. Under these conditions, Rim20p and Bro1p spatially segregate, at least partially, in the endosomal system (12). This led to the proposal that alkaline ambient pH specifies an endosomal domain identity that favours the assembly of pH signalling complexes as opposed to MVB complexes (12). That would solve the conundrum as far as the two apparently mutually exclusive pathways (MVB and pH signalling) are concerned but does not provide a satisfactory explanation for the ability of two Bro1-domain-containing proteins in the pH signalling ‘domain’ to interact with the same partner.

*Aspergillus nidulans*PalF is a pH signalling-dedicated arrestin-like protein interacting with the cytosolic tail of the 7-TMD receptor PalH. Under alkaline pH conditions, PalF is phosphorylated and ubiquitinated (10). Functional GFP-tagged PalC localizes to punctate structures at or near the plasma membrane (this work) also under alkaline conditions. Although both PalF ubiquitination and PalC-GFP localization are PalH dependent, PalC plays its role downstream of PalF phosphorylation/ubiquitination (10). Thus, an attractive but as yet untested possibility is that PalC is recruited to its plasma membrane-associated localization through phosphorylated and ubiquitinated PalF.

Signal-dependent ubiquitination of arrestin-like PalF strongly indicates that pH signal transduction involves endocytic trafficking. However, PalC-GFP cortical structures cannot be endosomes as they are closely associated with the plasma membrane and are static (Movie S1), whereas *A. nidulans*early endosomes are cytosolic and, most notably, highly motile (see below and Movie S2). One possibility is that PalC cortical structures represent a plasma membrane subdomain where endocytosis of the pH signalling complexes takes place. PalH-GFP localization to the plasma membrane is assisted by PalI (A. M. Calcagno, S. Negrete-Urtasun, H. N. A. and M. A. P., unpublished data), but its detection requires overexpression, which results in more or less uniform labelling of the plasma membrane that would almost certainly make colocalization experiments uninformative. The subcellular localization of PalA has not yet been determined but, as noted above, its *S. cerevisiae*orthologue Rim20p does localize to endosomes (12). Thus, it seems plausible that pH signalling complexes characterized as containing either PalC or PalA also segregate spatially.

By means of its ability to localize to cortical structures and to bind Vps32, PalC has the expected features of a link between the upstream and the downstream pH signalling complexes. We note that a dynamic spatiotemporal localization of pH signalling proteins to pH signalling complexes whose protein composition would change following endocytosis could account for the observations reported here. We hypothesize that one role of PalC in its cortical location at or in the proximity of the plasma membrane is determining pH signalling identity on membranes and that this role involves the recruitment of the endosomal Vps20/Vps32 ESCRT-III coat, which PalC binds. Subsequent maturation of these ‘pH signalling membranes’ following endocytosis would result in Vps32-mediated recruitment of PalA to these endosomal domains with release of PalC. This spatiotemporal localization of protein components to heterooligomeric complexes would resemble the maturation of endocytic patches during clathrin-dependent internalization (59).

Recruitment of ESCRT proteins to the plasma membrane is not without precedent. It is well established that the Bro1 domain protein and Vps32 interactor Alix and ESCRTs are involved in retroviral budding at the plasma membrane (see 60, for a review). A crucial test of the above hypothetical model for pH regulation in *Aspergillus*would be determining whether a proportion of fully functional Vps32 expressed at physiological levels can be recruited to a cortical subcellular localization in addition to endosomes. One rigorous, recent study has used quantitative electron microscopy to demonstrate that a proportion of Alix and of the ESCRT proteins Tsg101/Vps23, Hrs/Vps27 and Vps4B associates with the plasma membrane (61).

In *Ustilago maydis*(62,63) and *A. nidulans*(42 and this work), early endosomes move bidirectionally on microtubule tracks. These data strongly suggest that this motility is a conserved feature of early endosomes of filamentous fungi. Vps32 binds to endosomal membranes. We address here the subcellular localization of fluorescent protein-tagged Vps32 in *A. nidulans*. Our analysis demonstrates that Vps32-GFP/mRFP localizes to relatively static cytosolic structures possibly representing mature endosomes/small vacuoles and, most notably, to bidirectionally moving cytosolic specks. These moving specks very likely represent early endosomes moving on microtubules. Microtubules are often subcortical and eventually contact the cell cortex (45,46). Indeed, some moving endosomes pass very near the cortex (Movie S3). Thus, one hypothetical possibility would be that transient recruitment of Vps32-containing moving endosomes to the proximity of the plasma membrane facilitates interaction between pH signalling proteins at the plasma membrane and those on endosomes.

Metazoan cells usually display a greater genetic complexity than fungi, often involving gene duplication events leading to multigene families and tissue-specific function. The Bro1-domain-containing protein Alix, a protein playing multiple roles in processes apparently as diverse as MVB sorting and inward vesicle budding, apoptosis, enveloped virus budding, regulation of receptor tyrosine kinase endocytosis or cell adhesion (see 64, for a review), is atypical in that it has two fungal homologues, BroA/Bro1p and PalA/Rim20p. Our identification of PalC as the third ascomycete Bro1-containing Vps32-binding protein (and the second involved in pH regulation) underscores one positive-acting regulatory role of endosomal ESCRT coats, which adds a reasonably well characterized and genetically amenable ascomycete signalling pathway to the list of ESCRT functions not involving MVB body sorting (60).

## Materials and Methods

### Aspergillus nidulans techniques

*Aspergillus nidulans*strains carried markers in standard use (65). MAD1112 *yA2 areA^r^58 inoB2 palC4 argB2*was used as recipient for site-directed integration at the *argB*locus of *alcA*^p^-driven transgenes. MAD782 *yA2 pabaA1 pyrG89*was used for gene replacement of *palC*by the *palC::GFP*allele. Transformation (66) and phenotypic testing of pH regulatory phenotypes in *alcA*^p^-PalC-GFP wild type and mutant strains (67) have been described. For Western blot analysis, strains containing *alcA*^p^-driven transgenes were cultured for 16 h at 30°C in synthetic complete medium containing 0.05% (w/v) glucose as carbon source. Mycelia were collected by filtration and transferred to the same medium with either 1% (v/v) ethanol or 2% glucose as carbon sources, corresponding to inducing and repressing conditions, respectively, and incubated for an additional 3 h before proceeding to protein extraction. Strains carrying *alcA^p^*::*palC-GFP*or gene-replaced *palC-GFP*in combination with *palA34*, *palH72*, *didBΔ*and *rab5Δ*mutations and with gene-replaced *abpA-mRFP*were constructed by meiotic crossing. *abpA-mRFP*, *didBΔ*and *rab5Δ*alleles will be reported elsewhere.

### Western blot analyses

*Aspergillus nidulans*mycelia collected on Miracloth (Calbiochem) were pressed dry, frozen in liquid nitrogen and lyophilized overnight. Protein was extracted as described (67). Fifty-microgram protein samples were resolved in 10% polyacrylamide gels before electrotransfer to nitrocellulose filters, which were reacted with either Roche anti-GFP mouse monoclonal antibody cocktail (1/1000, clones 7.1 and 13.1) or, for loading controls, with mouse anti-actin (1/5000, clone C4, ICN Biomedicals Inc.). Peroxidase conjugated goat anti-mouse IgG immunoglobin (Jackson) was used as secondary antibody at 1/4000. Vps32-mRFP was detected using an anti-mRFP chicken polyclonal antibody (Clontech) (1/5000) and peroxidase-coupled anti-chicken (Clontech) IgG immunoglobulins (1/10 000) as secondary antibody.

Yeast extracts for Western blot analyses of GAL4_AD_ protein fusions in two-hybrid assays were made as described (68). Protein fusions were revealed with rat anti-hemagglutinin (anti-HA) peptide epitope antibody (clone 3F10, Roche) followed by secondary immunodetection using peroxidase-coupled goat anti-rat IgG (Southern Biotechnology, 1/4000). Actin was revealed as above.

### Plasmids

Characterization of *A. nidulans*genes encoding Vps25, Vps20, Vps32, Vps2, Vps24, Vps4 and DidB^Did2^ will be described elsewhere. Their complete coding regions were obtained from complementary DNA (cDNA) libraries and, like that of PalC, cloned into pGBKT7 and pACT2 as *NcoI*-*BamHI*fragments excepting Vps32, which was cloned as a *BamHI*fragment. *palC*cDNAs carrying mutations leading to Pro439Phe, Arg442His, Arg442Ala, Arg442Δ and Tyr451Ala changes and C-terminal truncating mutations were obtained after mutagenic polymerase chain reaction (PCR) and cloned, for two-hybrid experiments, in pACT2 as *NcoI*-*EcoRI*fragments. For pull downs, plasmids expressing wild type and mutant GST-PalC fusion proteins were obtained after cloning the corresponding cDNAs in pGEX-2T as *BamHI*-*EcoRI*fragments. N-zz-tagged fusion proteins used as preys in pull downs were expressed from pEQ80zz (69), where the corresponding cDNAs were cloned as *NcoI*-*BamHI*fragments. Plasmids driving expression of wild type and mutant (C tagged) PalC-GFP and wild-type Vps32-GFP under the control of the *alcA*^p^ were obtained by in-frame subcloning of *palC*and *Vps32*into a pALC-*argB*-*Bgl*II derivative expressing GFP (70). The construction of a similar plasmid expressing GFP-Rab5 will be described elsewhere. *palC*mutations were introduced as above. Plasmid pVps32-mRFP, which drives expression of Vps32-mRFP under the control of the *Vps32*promoter, was constructed by cloning into TOPO 2.1, a DNA fragment made by fusion PCR as described (41). The plasmid contains 622 bp of the *Vps32*promoter region followed by the *Vps32*gene sequence fused in frame to mRFP and by an *Aspergillus fumigatus*DNA fragment containing the selectable *pyrG*marker

### Characterization of recombinant A. nidulans strains

Transformants carrying single copy integration events of pALC-*argB*-*Bgl*II *PalC-GFP*, *Vps32-GFP, GFP-Rab5*and *GFP*derivatives at the *argB*locus were identified by Southern blot analysis as described (51,70). All these strains were shown to express GFP or GFP fusion proteins of the expected size by Western blot analyses. Strains in which *palC*was replaced by *palC*-GFP were constructed using the GA-5–GFP–Af-*pyrG*cassette (obtained from Dr S. Osmani) following Yang et al.(41). Several independent transformants, of which two were chosen for further analyses, showed the expected gene replacement as determined by Southern blots using a *palC*probe and by long-distance PCR (Expand Long Template PCR Kit, Roche) using flanking primers. These transformants expressed a GFP fusion protein of the expected size, as determined by Western blotting. Strains carrying single copy integration events of pVps32-mRFP were identified by Southern blotting and shown, by Western blot analysis, to express a Vps32-mRFP fusion protein of the expected size.

### Two-hybrid assays

Complementary DNA versions of bait and prey genes were inserted in frame into pGBKT7 and pACT2 vectors (Clontech). Appropriate combinations of bait and prey plasmids were transformed into *S. cerevisiae*AH109 (*MAT*a, *trp1-901*, *leu2-3*, *112*, *ura3-52*, *his3-200*, *gal4Δ*, *gal80Δ*, *LYS2::GAL1*_UAS_-*GAL1_TATA_*-*HIS3*, *GAL2_UAS_-GAL2_TATA_-ADE2*, *URA3::MEL1_UAS_-MEL1 _TATA_-lacZ*) and Y187 (*MATα*, *ura3-52*, *his3-200*, *ade2-101*, *trp1-901*, *leu2-3*, *112*, *gal4Δ*, *met*^–^, *gal80Δ*, *URA3::GAL1_UAS_-GAL1_TATA_-lacZ*) strains. AH109 primary transformants were patch inoculated onto -Leu, -Trp and -Leu, -Trp, -His, -Ade [quadruple selection (QSM)] synthetic dextrose media. Positive interactions were revealed by growth on QSM after 2 days of incubation at 30°C. Y187 primary transformants were patch inoculated onto -Leu, -Trp plates that were incubated for 2 days at 30°C before proceeding with qualitative β-galactosidase lift filter assays (71), using X-gal as substrate. Quantitative β-galactosidase assays in cells permeabilized with a mixture of dry ice and ethanol followed the Clontech Yeast Protocol Handbook (Clontech Laboratories Inc.). Beta-galactosidase activities (in Miller units) represent the average of five independent clones each assayed in triplicate.

### Pull-down assays

Proteins were expressed in *Escherichia coli*for 24 h at 20°C after induction with 0.1 mm isopropyl β-d-1-thiogalactopyranoside (IPTG). Cells were concentrated fivefold in BB50 buffer (50 mm Tris–HCl pH 7.5, 50 mm NaCl and 2 mm MgCl_2_) containing one tablet of Roche’s complete ethylenediaminetetraacetic acid (EDTA)-free protease inhibitor cocktail per 10 mL, lysed in a French Press and the resulting protein extracts clarified by centrifugation for 15 minutes at 20,000 ×***g***and 4°C. Bait and prey extracts (600 μg protein each) were mixed in 800 μL of binding buffer (10 mm Tris–HCl pH 8.0, 1 mm EDTA, 5 mm DTT, 0.5% Triton-X-100 and, unless otherwise indicated, 200 mm NaCl) and rotated for 3 h at 4°C in Handee-Spin Columns (Pierce) before addition of 25 μL of 50% (v/v) glutathione–Sepharose beads equilibrated in binding buffer and incubation at 4°C for a further 2 h. Beads were washed six times with binding buffer before elution of bound proteins in Laemmli sample buffer. Samples were run in SDS–polyacrylamide gels and stained with Coomassie. Pull downs from *A. nidulans*cell extracts containing GFP or GFP fusion proteins were made using 2 mg of protein incubated with the protein baits in binding buffer in the presence of the above inhibitor cocktail supplemented with Pefabloc (1 mm), leupeptin (0.3 μg/mL), pepstatin (0.3 μg/mL) and MG132 (10 μm).

### Yeast methods

*palC*(cDNA version) and *YGR122w*(amplified from yeast genomic DNA) were subcloned into pRS416 (72) and transformed into BY4742 (*MATα*, *his3Δ1*, *leu2Δ0*, *lys2Δ0*and *ura3Δ0*) using minus uracil selection. Lithium chloride was added to yeast extract-peptone-dextrose (YPD) plates at 200 mm final concentration. Plates were incubated at 30°C for 3 days.

### Microscopy

Germlings of strains carrying *alcA*^p^-driven wild type and mutant PalC-GFP transgenes and the GFP control were cultured on the surface of glass coverslips submerged in 2.5 mL of appropriately supplemented ‘watch’ minimal medium adjusted to acidic pH with 25 mm NaH_2_PO_4_(42), containing 1% (v/v) ethanol as sole carbon source. After 18 h at 25°C, germlings were transferred to the same medium containing 2% (w/v) glucose (to repress the *alcA*^p^), adjusted to either acidic (with 25 mm NaH_2_PO_4_, pH 5.2–5.3), neutral (with 12.5 mm NaH_2_PO_4_ plus 12.5 mm Na_2_HPO_4_, pH ∼7.0) or alkaline (25 mm Na_2_HPO_4_, pH 8.2–8.3) conditions for an additional 30 minutes before being mounted and imaged. MAD1373, carrying the *palC::GFP*gene replacement, was cultured under acidic conditions and transferred to acidic, neutral or alkaline pH media adjusted as above, using for all conditions 1% (v/v) glucose as carbon source. Microscopy was carried out using a Nikon E-600 upright epifluorescence microscope equipped with a 100× 1.40 NA plan apochromat objective. Green and red fluorescence were observed using B-2A and G-2A Nikon filter combinations and recorded with an Orca-ER camera (Hamamatsu) driven by Metamorph (Universal Image Co.) Contrast of 12-bit images was improved using either Metamorph or Wasabi 1.5 (Hamamatsu Photonics GmbH) software before their conversion to 8-bit format. PalC-GFP punctate structures were counted in at least 20 germlings per strain and pH condition and normalized to the germling length. Data are given as average number of punctate structures per germling and micron, with standard deviations indicated. FM-64 load-and-chase experiments were performed as described (42). Time-lapse microscopy was carried out using the above Nikon E-600 equipment or a Leica DMI-6000 B inverted microscope equipped with Leica GFP and TX2 filter cubes and an HCX 63× 1.40 NA plan apochromat objective. For this inverted microscope, Lab-Tek incubation chambered coverglasses (Nunc International) were used. Room temperature was adjusted to 26–28°C. Images were adquire with ORCA-ER cameras using the ‘stream acquisition’ module of MetaMorph (Universal Imaging). Watch minimal medium with 0.1% fructose used as sole carbon source for low-level expression of Vps32-GFP.

## Supplementary Materials

Figure S1 **Two-hybrid interactions on which the scheme in Figure 3C is based.** Positive interactions among the indicated baits (fusion proteins to the GAL4 DNA-binding domain) and preys (fusion proteins to the GAL4 activation domain) are revealed here by growth of *Saccharomyces cerevisiae* AH109 strains transformed with the corresponding plasmids on QSM (-Trp, -Leu, -His, -Ade) medium. Growth on SD -Trp, -Leu indicates the presence of bait and prey plasmids.

Figure S2 **pH regulatory phenotype of strains carrying *alcA::PalC-GFP* transgenes, whose expression is strongly induced on ethanol and repressed on glucose.** The acidity-mimicking loss of function mutation *palC4* present in all strains results in increased sensitivity to molybdate, increased resistance to neomycin and prevents growth on alkaline pH media. Complementation is indicated by increased tolerance to molybdate, increased sensitivity to neomycin and growth on alkaline pH media. Note that residual expression of the wild-type transgenes under repressing conditions results in slightly improved molybdate resistance and permits growth on alkaline pH plates. This was not seen for any of the mutant transgenes.

Figure S3 Control experiment in which GFP alone was expressed under the control of the *alcA^p^* before shifting cells to acidic or alkaline conditions, as indicated.

Figure S4 **Amino acid sequence alignment involving C-terminal regions of filamentous fungal and yeast PalC proteins, including *Saccharomyces cerevisiae* YGR122w and *Ashbya gossypii* AAR081c.** Conserved residues (according to the Blom62 matrix) are shaded in blue (dark, intermediate and light blue indicating 100, 80 and 60% conservation, respectively). The positions of single-residue substitutions and deletion preventing interaction with Vps32 as well as those of the truncations present in *palC131* and *palC159* mutant protein products are indicated. Bars, 5 μm.

Figure S5 **The lithium hypersensitivity phenotype resulting from deletion of *Saccharomyces cerevisiae YGR122w*, the likely yeast orthologue of PalC.** Yeast strains transformed with the indicated plasmids were grown to saturation on synthetic dextrose medium without uracil and serially diluted samples were plated on YPD or YPD containing 200 mM LiCl. Cell densities decrease from left to right. The lithium hypersensitivity phenotype is prevented by expression of YGR122w but not by PalC.

Movie S1 Time-lapse microscopy of cortical PalC-GFP structures over a 2-minute period. Frames were taken every 5 seconds using a 1-seconds exposure time. Time is in minutes:seconds:milliseconds.

Movie S2 Time-lapse microscopy of FM4-64 labelled endosomes. Frames were taken approximately every 0.1 seconds. Time is in seconds:milliseconds.

Movie S3 Time-lapse microscopy of Vps32-GFP-labelled endosomes. Frames were taken approximately every 0.5 seconds with a 2 × 2 binning.
