# Supplementary figures and images for: PalC, One of Two Bro1 Domain Proteins in the Fungal pH Signalling Pathway, Localizes to Cortical Structures and Binds Vps32
Source: Traffic. 2007 Aug 13;8(10):1346–64. doi: 10.1111/j.1600-0854.2007.00620.x (PMC2171039; doi:10.1111/j.1600-0854.2007.00620.x)

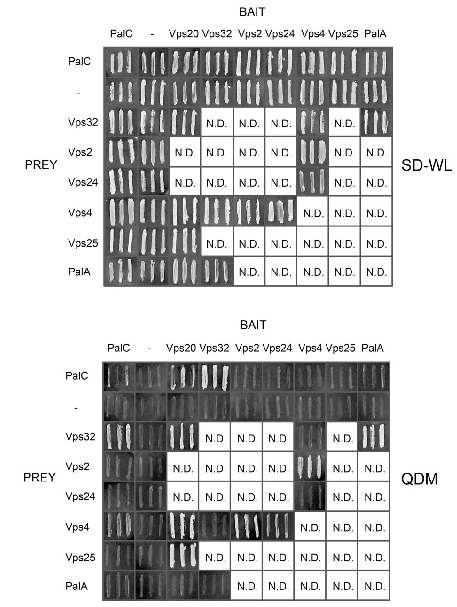

Supplement: Figure S1 — Two-hybrid interactions on which the scheme in Figure 3C is based. Positive interactions among the indicated baits (fusion proteins to the GAL4 DNA-binding domain) and preys (fusion proteins to the GAL4 activation domain) are revealed here by growth of Saccharomyces cerevisiae AH109 strains transformed with the corresponding plasmids on QSM (-Trp, -Leu, -His, -Ade) medium. Growth on SD -Trp, -Leu indicates the presence of bait and prey plasmids. [file tra0008-1346-SD1.tif]

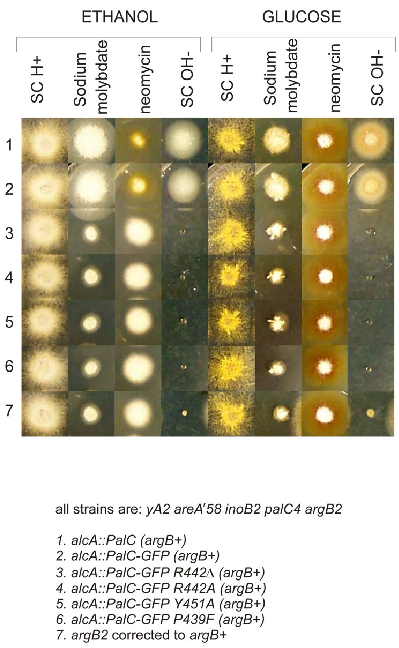

Supplement: Figure S2 — pH regulatory phenotype of strains carrying alcA::PalC-GFP transgenes, whose expression is strongly induced on ethanol and repressed on glucose. The acidity-mimicking loss of function mutation palC4 present in all strains results in increased sensitivity to molybdate, increased resistance to neomycin and prevents growth on alkaline pH media. Complementation is indicated by increased tolerance to molybdate, increased sensitivity to neomycin and growth on alkaline pH media. Note that residual expression of the wild-type transgenes under repressing conditions results in slightly improved molybdate resistance and permits growth on alkaline pH plates. This was not seen for any of the mutant transgenes. [file tra0008-1346-SD2.tif]

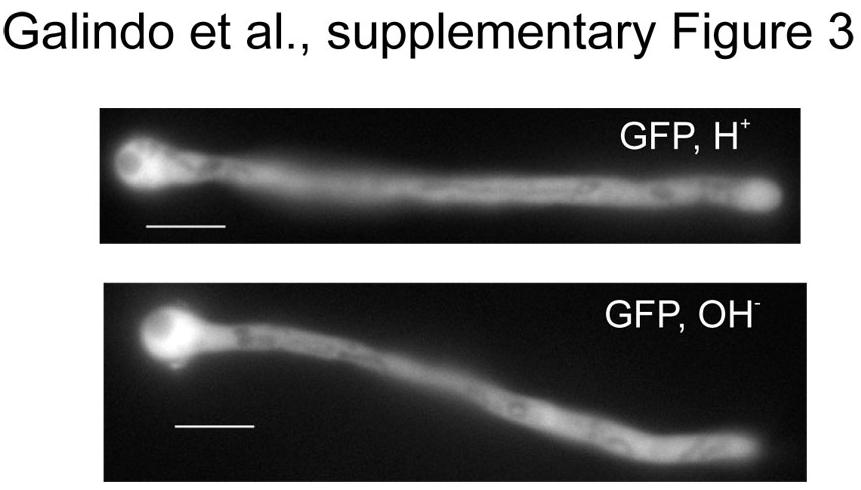

Supplement: Figure S3 — Control experiment in which GFP alone was expressed under the control of the alcAp before shifting cells to acidic or alkaline conditions, as indicated. [file tra0008-1346-SD3.tif]

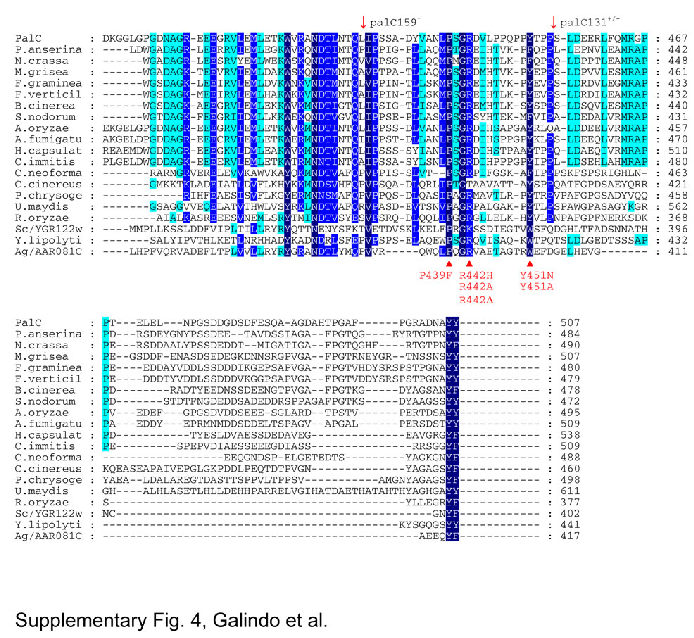

Supplement: Figure S4 — Amino acid sequence alignment involving C-terminal regions of filamentous fungal and yeast PalC proteins, including Saccharomyces cerevisiae YGR122w and Ashbya gossypii AAR081c. Conserved residues (according to the Blom62 matrix) are shaded in blue (dark, intermediate and light blue indicating 100, 80 and 60% conservation, respectively). The positions of single-residue substitutions and deletion preventing interaction with Vps32 as well as those of the truncations present in palC131 and palC159 mutant protein products are indicated. Bars, 5 μm. [file tra0008-1346-SD4.tif]

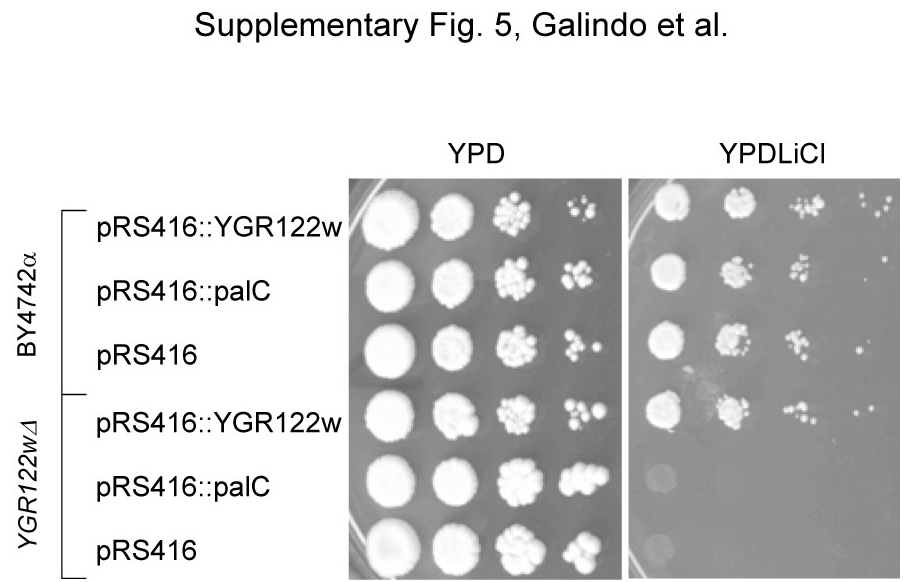

Supplement: Figure S5 — The lithium hypersensitivity phenotype resulting from deletion of Saccharomyces cerevisiae YGR122w, the likely yeast orthologue of PalC. Yeast strains transformed with the indicated plasmids were grown to saturation on synthetic dextrose medium without uracil and serially diluted samples were plated on YPD or YPD containing 200 mM LiCl. Cell densities decrease from left to right. The lithium hypersensitivity phenotype is prevented by expression of YGR122w but not by PalC. [file tra0008-1346-SD5.tif]
